# Dr. Albert J. Browne

**Published:** 1915-03

**Authors:** 


**Dr. Albert J. Browne,** Berkshire 1865, for many years physician to the Masonic Home at Utica, died Feb.. . The remains were buried at Newport. (Berkshire Medical College was organized in 1824 at Pittsfield, Mass., was the medical department of Williams College, and became extinct in 1867).

